# Anti-Inflammatory Effect of Quercetin on RAW 264.7 Mouse Macrophages Induced with Polyinosinic-Polycytidylic Acid

**DOI:** 10.3390/molecules21040450

**Published:** 2016-04-04

**Authors:** Young-Jin Kim, Wansu Park

**Affiliations:** Department of Pathology, College of Korean Medicine, Gachon University, Seong-nam 13120, Korea; godsentry@naver.com

**Keywords:** quercetin, dsRNA, inflammation, macrophages, nitric oxide, cytokine, calcium, STAT

## Abstract

Quercetin (3,3′,4′,5,6-pentahydroxyflavone) is a well-known antioxidant and a flavonol found in many fruits, leaves, and vegetables. Quercetin also has known anti-inflammatory effects on lipopolysaccharide-induced macrophages. However, the effects of quercetin on virus-induced macrophages have not been fully reported. In this study, the anti-inflammatory effect of quercetin on double-stranded RNA (dsRNA)-induced macrophages was examined. Quercetin at concentrations up to 50 μM significantly inhibited the production of NO, IL-6, MCP-1, IP-10, RANTES, GM-CSF, G-CSF, TNF-α, LIF, LIX, and VEGF as well as calcium release in dsRNA (50 µg/mL of polyinosinic-polycytidylic acid)-induced RAW 264.7 mouse macrophages (*p* < 0.05). Quercetin at concentrations up to 50 μM also significantly inhibited mRNA expression of signal transducer and activated transcription 1 (STAT1) and STAT3 in dsRNA-induced RAW 264.7 cells (*p* < 0.05). In conclusion, quercetin had alleviating effects on viral inflammation based on inhibition of NO, cytokines, chemokines, and growth factors in dsRNA-induced macrophages via the calcium-STAT pathway.

## 1. Introduction

Immunity is essential for life and innate immunity provides the first line of response to invading pathogens [1]. Inflammation is a primordial response that protects against infection and restores damaged tissue to its normal physiological functioning [2]. A recent study reported that it is becoming more important to maintain inflammatory homeostasis and to regulate the immune system [3]. Inflammation is an innate immune response by various immune cells, such as macrophages, to protect against harmful stimuli, such as viruses and bacteria [4]. During the inflammatory reaction, macrophages produce inflammatory mediators, such as nitric oxide (NO), cytokines, and growth factors. Calcium is released by macrophages during inflammatory processes. Therefore, modulating macrophage-mediated inflammatory responses is important for creating a new therapeutic approach against inflammatory diseases [5].

Viruses can induce tissue necrosis and inflammation and viral infections activate the immune response, trigger inflammatory diseases, and promote cancer growth [6]. dsRNA, which is recognized by Toll-like receptor-3, induces the production of inflammatory mediators, such as NO, interleukin (IL)-6, and tumor necrosis factor (TNF)-α, in macrophages; polyinosinic-polycytidylic acid (poly(I:C)) is considered a synthetic analog of dsRNA [7]. dsRNA activates nuclear factor kappa beta and the calcium signaling pathway [8].

**Figure 1:**
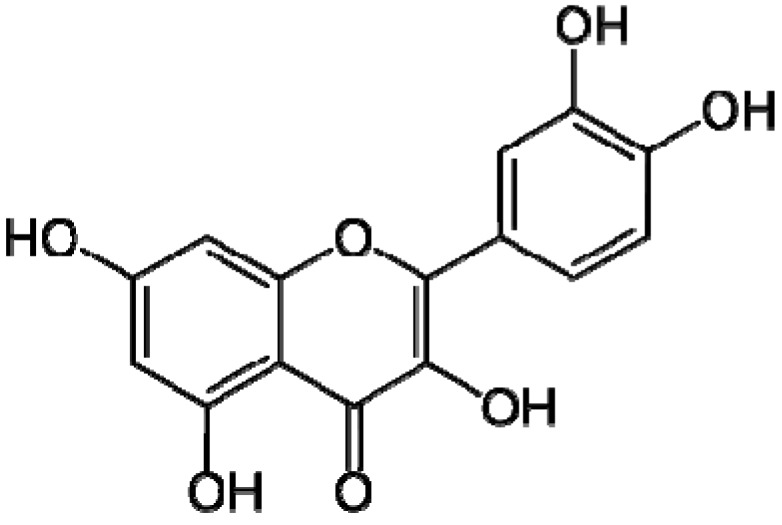
Chemical structure of quercetin.

Quercetin (3,3′,4′,5,6-pentahydroxyflavone, Figure 1) is a flavonol found in many fruits, vegetables, and leaves [9]. The biological activities of quercetin have been extensively demonstrated in various experimental models. For example, Liao and Lin have reported that quercetin significantly increased IL-10 secretions by peritoneal macrophages of the lipopolysaccharide (LPS)-induced septic mice [10]; Gardi *et al.* have reported that quercetin lowered levels of IL-1β, C-reactive protein, and monocyte chemotactic protein-1 (MCP-1) in a rat model of adjuvant arthritis [11]; Guazelli *et al.* have reported that the oral administration of quercetin -loaded microcapsules decreases neutrophil recruitment, attenuates histological alterations, and reduces macroscopical damage, edema, and IL-1β and IL-33 production in a mouse model of acetic acid -induced colitis [12]. Recently, Chiow *et al.* have reported that quercetin could inhibit both mouse hepatitis virus and dengue virus (type 2) [13]. Wo *et al.* have reported that quercetin may exert its antiviral activity against different influenza virus strains (including H1N1 and H3N2) via interaction with viral hemagglutinin protein and then inhibit virus entry into the cell [14]. However, the effects of quercetin on poly(I:C)-induced macrophages have not been fully reported.

In the present study, quercetin at concentrations up to 50 μM significantly inhibited the production of various factors, as well as calcium release and mRNA expression of signal transducer and activated transcription 1 (STAT1) and STAT3 in poly(I:C)-induced RAW 264.7 mouse macrophages.

## 2. Results

### 2.1. Effect of Quercetin on NO Production and Intracellular Calcium Release

**Figure 2:**
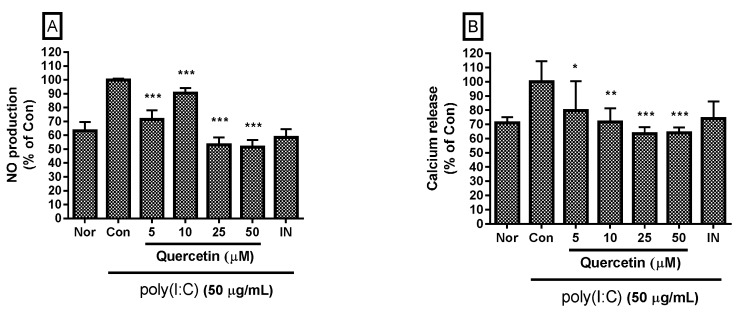
Effect of quercetin on nitric oxide (NO) production (**A**) and calcium release (**B**) by dsRNA-induced RAW 264.7 mouse macrophages. After 24 h treatment, NO production was measured by the Griess reaction assay. Calcium release was measured with the Fluo-4 calcium assay after an 18 h treatment. The normal group (Nor) was treated with media only. The control group (Con) was treated with 50 µg/mL of the dsRNA synthetic analog polyinosinic-polycytidylic acid (poly(I:C)) alone. IN, indomethacin (0.5 µM). Values are mean ± standard deviation of three independent experiments. * *p* < 0.05 *vs.* Con; ** *p* < 0.01; *** *p* < 0.001. Significant differences were examined using one-way analysis of variance test followed by Tukey’s multiple comparison test.

NO is a major inflammatory mediator during the immuno-inflammatory reaction. NO concentration in culture medium can be determined by the Griess reaction. In this study, RAW 264.7 mouse macrophages were incubated with poly(I:C) and/or quercetin for 24 h, and 100 µL of supernatant from each well was mixed with 100 µL Griess reagent in a 96-well plate to determine NO concentration. The results show that quercetin significantly inhibited excessive production of NO in the cells at concentrations of 5, 10, 25, and 50 µM (Figure 2). No association was found between quercetin concentration and NO level. The inhibitory effect of 50 µM quercetin stronger than that at 5 and 10 µM. Therefore, quercetin concentrations of 10, 25, and 50 µM were chosen for the multiplex cytokine assay, and concentrations of 25 and 50 µM were chosen for the STAT mRNA expression evaluation.

Intracellular calcium release is related with many cellular processes including the inflammatory reaction. Calcium release from RAW 264.7 mouse macrophages can be determined by the Fluo-4 assay. Because calcium is involved in a signaling pathway, the cells were incubated with molecules for 18 h in this study. After incubating the cells with poly(I:C) and/or quercetin for 18 h, the medium was removed, and the cells were incubated with the Fluo-4 dye loading solution. After incubation, fluorescence intensity of each well was determined spectrofluorometrically with excitation and emission filters of 485 nm and 535 nm, respectively. The data show that quercetin inhibited significantly calcium release from poly(I:C)-induced RAW 264.7 mouse macrophages at concentrations of 5, 10, 25, and 50 µM (Figure 2). No significant association was found between quercetin concentration and intracellular calcium level.

The NO production and calcium release results are shown as a percentage relative to the control group. Indomethacin was used as a positive control.

### 2.2. Effect of Quercetin on Cytokine Production

**Figure 3:**
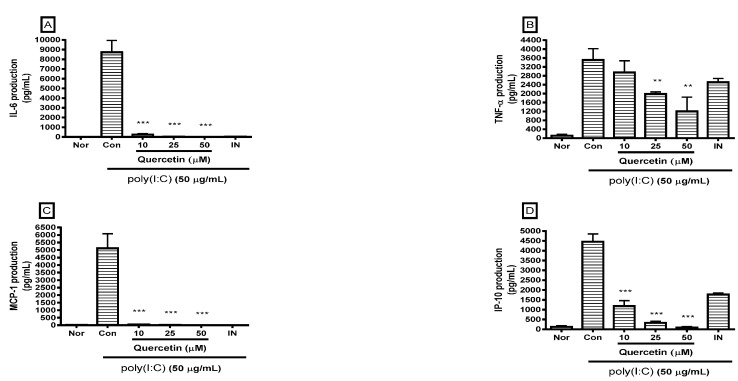

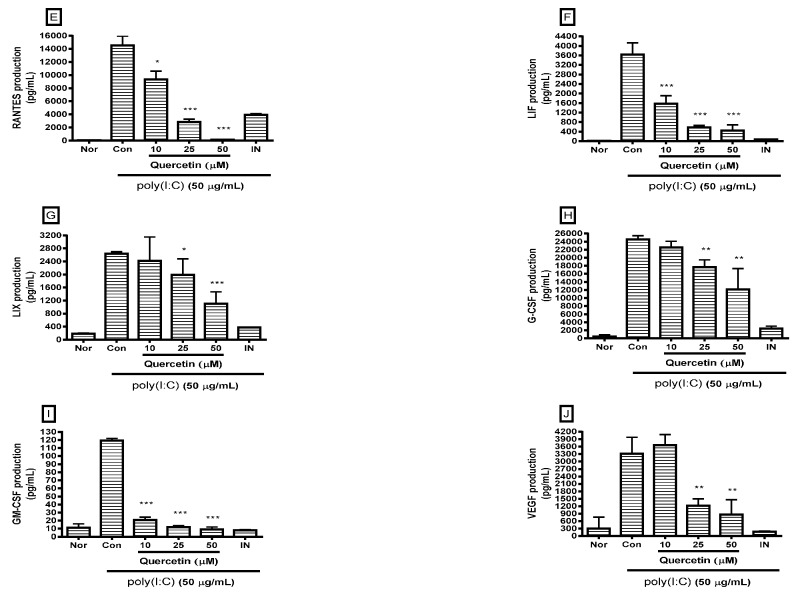
Effect of quercetin on production of cytokines, such as interleukin (IL)-6 (**A**); tumor necrosis factor (TNF)-α (**B**); monocyte chemoattractant protein-1 (MCP-1) (**C**); interferon gamma-induced protein-10 (IP-10) (**D**); RANTES (**E**), leukemia inhibitory factor (LIF) (**F**); lipopolysaccharide-induced CXC chemokine (LIX) (**G**); granulocyte-colony stimulating factor (G-CSF) (**H**); granulocyte macrophage-colony stimulating factor (GM-CSF) (**I**); and vascular endothelial growth factor (VEGF) (**J**) in dsRNA-induced RAW 264.7 mouse macrophages. Flourescene intensity of each cytokine in the culture medium was measured by a multiplex bead-based cytokine assay after the 24 h incubation. The normal group (Nor) was treated with media only. The control group (Con) was treated with 50 µg/mL of the dsRNA synthetic analog polyinosinic-polycytidylic acid (poly(I:C)) alone. IN, indomethacin (0.5 µM). Values are mean ± standard deviation of three independent experiments. * *p* < 0.05 *vs.* Con; ** *p* < 0.01; *** *p* < 0.001. Significant differences were examined using one-way analysis of variance test followed by Tukey’s multiple comparison test.

Cytokines are a distinct category of small (5–20 kDa) proteins that are important in inflammation and cell signaling. Cytokine production in culture medium was determined by the multiplex cytokine assay. After incubating the cells with poly(I:C) and/or quercetin for 24 h, the cytokines released from the cells were measured in cell culture supernatants using the Luminex assay based on xMAP technology. The result show that quercetin significantly reduced excess production of IL-6, TNF-α, MCP-1, interferon gamma-induced protein-10 (IP-10), regulated on activation, normal T cell expressed and secreted (RANTES), leukemia inhibitory factor (LIF), lipopolysaccharide-induced CXC chemokine (LIX), granulocyte-colony stimulating factor (G-CSF), granulocyte macrophage-colony stimulating factor (GM-CSF), and vascular endothelial growth factor (VEGF) in poly(I:C)-induced RAW 264.7 mouse macrophages (Figure 3).

### 2.3. Effect of Quercetin on STAT1 and STAT3 mRNA Expression

**Figure 4:**
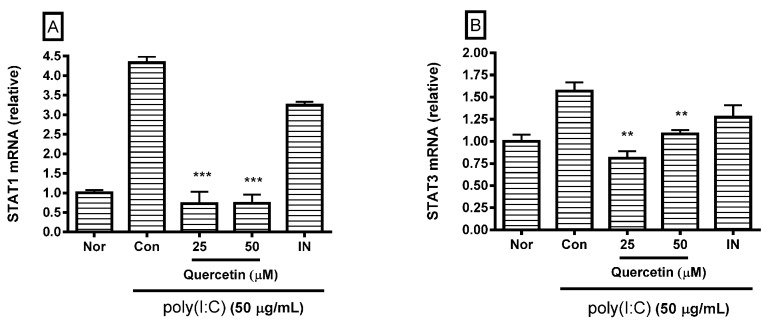
Effect of quercetin on signal transducer and activated transcription 1 (STAT1) (**A**) and STAT3 mRNA expression (**B**) in dsRNA-induced RAW 264.7 mouse macrophages. After an 18 h treatment, STAT1 and STAT3 mRNA expression was measured by real-time reverse-transcription polymerase chain reaction assay. STAT1 and STAT3 mRNA were normalized to GAPDH mRNA. The normal group (Nor) was treated with media only. The control group (Con) was treated with 50 µg/mL of the dsRNA synthetic analog polyinosinic-polycytidylic acid (poly(I:C)) alone. IN, indomethacin (0.5 µM). Values are the mean ± standard deviation of three independent experiments. ** *p* < 0.01 *vs.* Con; *** *p* < 0.001. Significant differences were examined using one-way analysis of variance test followed by Tukey’s multiple comparison test.

Because STAT proteins are involved in a signaling pathway that activates macrophages, the cells were incubated with molecules for 18 h. After incubating the cells with poly(I:C) and/or quercetin for 18 h, real-time reverse-transcription polymerase chain reaction (RT-PCR) was performed to determine STAT1 (GenBank: NM_009283) and STAT3 (GenBank: NM_213659) mRNA expression. The GAPDH gene (GenBank: NM_001001303) was used for RNA normalization. The results show that quercetin significantly inhibited STAT1 and STAT3 mRNA expression in poly(I:C)-induced RAW 264.7 mouse macrophages at concentrations of 25 and 50 µM (Figure 4), indicating that quercetin modulates inflammatory reactions in poly(I:C)-induced macrophages via the calcium-STAT pathway.

## 3. Discussion

Inflammation in the immune system is essential to fight an infection and is a general term used to describe many diverse processes that tissues employ in response to infections by pathogens (such as bacteria, viruses, parasites, and fungi) and injuries caused by toxic molecules or physical damage such as burns or cuts [15].

Viral infections trigger anti-viral type I interferon responses by stimulating endosomal and cytosolic pattern recognition receptors, including Toll-like receptors, which are key initiators of inflammation [16]. Immunomodulatory approaches are being considered to combat a vast range of human and animal diseases including incurable viral diseases, cancers, autoimmune diseases, and inflammatory conditions [17]. Viral and bacterial infections exacerbate the immune system and increase morbidity of patients with chronic airways diseases; viral infections trigger an innate immune response through host detection of viral dsRNA via several different mechanisms, including activation of a member of the class of pattern recognition receptors, Toll-like receptor 3; lung inflammation associated with viral infections includes robust increase in cytokines, such as IL-1β, IL-6, IL-8, TNF-α, GM-CSF, IP-10, RANTES, and MIP-1α [18].

Among the many immuno-inflammatory leukocytes, macrophages and monocytes are of great importance [19]. Macrophages are found throughout all tissues and are a key component of the immune system. Recruitment of macrophages to specific sites is important for normal host defense [20], as they play an important role in inflammatory disease by releasing factors, such as NO, reactive oxygen species, inflammatory cytokines, chemokines, growth factors, and prostaglandin mediators involved in the immune response [21].

NO is a membrane-permeable signaling molecule involved in a broad array of biologic processes through its ability to modify proteins, lipids, and DNA and alter their functions and immunogenicity [22]. Appropriate levels of NO assist in mounting an effective defense against invading microbes, whereas the inability to generate NO results in serious, even fatal, susceptibility to infections. Furthermore, dysregulation or overproduction of NO has been implicated in the pathogenesis of many disorders, including atherosclerosis, neurodegenerative diseases, inflammatory autoimmune diseases, and cancers; therefore, the potential exists for NO to behave like a “double-edged” biological sword depending on it level. Thus, it is crucial to understand regulation of NO [23].

Some studies show that the regulation of inflammation is important for maintaining homeostasis [3]. Inflammation plays a critical defensive role in the human body, however, uncontrolled or aberrant inflammatory responses contribute to various acute and chronic inflammatory diseases [24]. Inflammation helps restore homeostasis after an insult but can be more damaging than the insult itself if uncontrolled, excessive, or prolonged [25]. For example, dysregulation of the inflammatory/immune responses is responsible for multiple organ failure in patients with sepsis, for which over expression of proinflammatory cytokines is a major mechanism [26]. It is well known that viral and bacterial infections contribute to the pathogenesis of severe sepsis, which is characterized by an overwhelming production of NO and proinflammatory cytokines, such as IL-6 [27]. Despite antibiotics, sepsis remains a clinical challenge, with high mortality rates and increasing prevalence [28]. The expression level of RANTES increases in lung tissue infected with influenza virus [29]. The robust, uncontrolled cytokine production, otherwise known as hypercytokinemia or a “cytokine storm”, is thought to initiate the severe systemic inflammatory response in various infections, such as those caused by influenza virus, cytomegalovirus, variola virus, severe acute respiratory syndrome coronavirus, and avian H5N1 influenza virus [30]. Thus, it is important to focus on therapeutics to modulate virus-induced hyperinflammation.

The poly(I:C)-induced activation of macrophages is regarded as an experimental model of viral inflammation *in vitro*. Although many studies on biological effects of quercetin have been reported, the effect of quercetin on poly(I:C)-induced macrophages has not been fully reported. The present study shows that quercetin may alleviate poly(I:C)-induced inflammation by decreasing levels of inflammatory mediators, such as NO, cytokines, chemokines, and growth factors, in RAW 264.7 mouse macrophages; quercetin is a candidate for modulating virus-hyperinflammation such as a cytokine storm.

Oxidative stress reduces endoplasmic reticulum (ER) calcium stores and increases intracellular calcium concentrations, resulting in ER stress-mediated STAT1 activation. Preapoptotic ER stress itself has proinflammatory effects in macrophages [31]. In the present study, quercetin significantly inhibited calcium release and STAT1 and STAT3 mRNA expression in poly(I:C)-induced RAW 264.7 mouse macrophages. Thus, quercetin might modulate poly(I:C)-induced macrophage activation via the calcium-STAT pathway.

Finally, the present study demonstrated that quercetin has anti-inflammatory effects related with inhibiting NO, IL-6, TNF-α, MCP-1, IP-10, RANTES, LIF, LIX, G-CSF, GM-CSF, and VEGF production in poly(I:C)-induced macrophages via the calcium-STAT pathway. Further study is needed to evaluate the clinical utility of quercetin for viral inflammation.

## 4. Materials and Methods

### 4.1. Materials

DMEM, fetal bovine serum, penicillin, streptomycin, PBS, and other tissue culture reagents were purchased from Gibco BRL (Grand Island, NY, USA). Quercetin, indomethacin, Griess reagent, and all other chemicals were purchased from Sigma-Aldrich (St. Louis, MO, USA).

### 4.2. Quantification of NO Production and Intracellular Calcium Release

NO concentration in culture medium was determined by the Griess reaction. Specifically, after incubating the cells with poly(I:C) and/or quercetin for 24 h, 100 µL of supernatant from each well was mixed with 100 µL Griess reagent in a 96-well plate. After a 15 min incubation at room temperature, optical density was determined at 540 nm with a microplate reader (Bio-Rad, Hercules, CA, USA) [32].

Calcium release from RAW 264.7 mouse macrophages was determined by the Fluo-4 assay. RAW 264.7 mouse macrophages in 96-well plates were incubated with poly(I:C) and/or quercetin for 18 h at 37 °C. Thereafter, the medium was removed, and the cells were incubated with 100 µL of the Fluo-4 dye loading solution (Molecular Probes, Eugene, OR, USA) for 30 min at 37 °C. After the incubation, fluorescence intensity in each well was determined spectrofluorometrically (Dynex, West Sussex, UK) with excitation and emission filters of 485 nm and 535 nm, respectively [33].

### 4.3. Multiplex Bead-Based Cytokine Assay

After 24 h treatment with poly(I:C) and/or quercetin, the cytokines released from treated cells were measured in cell culture supernatants using a Luminex assay based on xMAP technology. This assay was performed with Milliplex kits (Millipore, Billerica, MA, USA) and the Bio-Plex 200 suspension array system (Bio-Rad) as described previously [34,35,36]. Standard curves for each cytokine were generated using the kit-supplied reference cytokine samples.

### 4.4. RNA Isolation and Real Time RT-PCR Analysis

At the end of the 18 h incubation with poly(I:C) and/or quercetin, RAW 264.7 mouse macrophages were lysed and total RNA was isolated using the NucleoSpin RNA kit (Macherey-Nagel, Duren, Germany) according to the manufacturer’s instructions. RNA quantity and quality were confirmed using the Experion RNA StdSens Analysis kit (Bio-Rad) and Experion Automatic Electrophoresis System (Bio-Rad). cDNA was synthesized from 1 µg total RNA using the iScript cDNA Synthesis kit (Bio-Rad). Real-time RT-PCR was performed using the iQ SYBR Green Supermix (Bio-Rad). The GAPDH gene was used for RNA normalization. The analysis was performed on a Bio-Rad CFX 96 Real-Time PCR Detection System (Bio-Rad). Relative changes in gene expression were calculated using the comparative threshold cycle (Ct) method (Bio-Rad) [12]. The following primers were used: STAT1 (GenBank: NM_009283), sense: 5′-TGAGATGTCCCGGATAGTGG-3′, antisense: 5′-CGCCAGAGAGAAATTCGTGT-3′; STAT3 (GenBank: NM_213659), sense: 5′-GTCTGCAGAGT TCAAGCACCT-3′, antisense: 5′-TCCTCAGTCACGATCAAGGAG-3′; GAPDH (GenBank: NM_0010 01303), sense: 5′-AACCTGCCAAGTATGATGAC-3′, antisense: 5′-GGGAGTTGCTGTTGAAGT-3′.

### 4.5. Statistical Analysis

The results shown are summarized from three independent experiments and represent mean ± standard deviation. Significant differences were examined using one-way analysis of variance test followed by Tukey’s multiple comparison test with SPSS 11.0 software (SPSS Inc., Chicago, IL, USA). In addition, a correlation analysis was conducted to examine the relationships between quercetin concentration and the levels of NO and/or intracellular calcium. A *p*-value < 0.05 was considered significant.

## 5. Conclusions

In conclusion, this study demonstrated that quercetin has anti-inflammatory effects related with inhibiting NO, IL-6, TNF-α, MCP-1, IP-10, RANTES, LIF, LIX, G-CSF, GM-CSF, and VEGF production in poly(I:C)-induced macrophages via the calcium-STAT pathway. Further study is needed to evaluate the clinical utility of quercetin for viral inflammation.

## Abbreviations

The following abbreviations are used in this manuscript:

NO	nitric oxide
dsRNA	double-stranded RNA
TLR	Toll like receptor
poly(I:C)	polyinosinic-polycytidylic acid
LPS	lipopolysaccharide
IL	interleukin
TNF	tumor necrosis factor
MCP	monocyte chemotactic protein
IP-10	interferon inducible protein-10
LIX	lipopolysaccharide-induced CXC chemokine
LIF	leukemia inhibitory factor
G-CSF	granulocyte colony-stimulating factor
GM-CSF	granulocyte macrophage colony-stimulating factor
VEGF	vascular endothelial growth factor
STAT1	signal transducers and activators of transcription 1
STAT3	signal transducers and activators of transcription 3
Nor	normal
Con	control
ER	endoplasmic reticulum
